# Mobile tablets for real-time data collection for hospital-based birth defects surveillance in Kampala, Uganda: Lessons learned

**DOI:** 10.1371/journal.pgph.0000662

**Published:** 2022-06-24

**Authors:** Dennis Kalibbala, Ayoub Kakande, Robert Serunjogi, Dhelia Williamson, Daniel Mumpe-Mwanja, Joyce Namale-Matovu, Diana Valencia, Beatrice Nalwoga, Christine Namirembe, Joan Seyionga, Margaret Nanfuka, Sophia Nakimuli, Margaret Okwero Achom, Kenneth Mwambi, Philippa Musoke, Linda Barlow-Mosha

**Affiliations:** 1 Makerere University–Johns Hopkins University Research Collaboration (MUJHU), Kampala, Uganda; 2 Medical Research Council/Uganda Virus Research Institute and London School of Hygiene & Tropical Medicine Uganda Research Unit, Entebbe, Uganda; 3 US Centers for Disease Control and Prevention, Atlanta, GA, United States of America; 4 US Centers for Disease Control and Prevention, Kampala, Uganda; 5 Makerere University College of Health Sciences, Kampala, Uganda; National University of Singapore, SINGAPORE

## Abstract

Sustainable birth defects surveillance systems provide countries with estimates of the prevalence of birth defects to guide prevention, care activities, and evaluate interventions. We used free and open-source software (Open Data Kit) to implement an electronic system to collect data for a hospital-based birth defects surveillance system at four major hospitals in Kampala, Uganda. We describe the establishment, successes, challenges, and lessons learned from using mobile tablets to capture data and photographs. After intensive training, surveillance midwives collected data using Android tablets with inbuilt logic checks; another surveillance midwife checked the quality of the data in real-time before data were securely uploaded onto a local server. Paper forms were used when needed as a backup for the electronic system. We experienced several challenges implementing the surveillance system, including forgotten passwords, unstable network, reduced tablet speed and freezing, loss of touch-screen sensitivity, decreased battery strength, and repetitive extensive retraining. We addressed these challenges by backing up and removing all photos from the tablet, uninstalling irrelevant applications to the study to increase storage space and speed, and monitoring and updating the system based mainly on feedback from the midwives. From August 2015 to December 2018, surveillance midwives documented information on 110,752 births at the participating hospitals. Of these, 110,573 (99.8%) were directly entered into the electronic data system and 179 (0.2%) were captured on paper forms. The use of mobile tablets for real-time data collection was successful in a hospital-based birth defects surveillance system in a resource-limited setting. Extensive training and follow-up can overcome challenges and are key to preparing staff for a successful data collection system.

## Introduction

Most hospitals in resource-limited settings do not have electronic medical record systems and typically collect medical information using paper-based forms. Data collection for research purposes in sub-Saharan Africa has traditionally been paper-based because the software, hardware, and internet connection for electronic data capture have been expensive and not widely available [1]. However, paper-based data collection systems have several potential limitations, including blank responses, invalid data entries, and transcription errors during data entry [2, 3]. Fortunately, software and hardware for electronic data capture systems are becoming affordable and more widely available [1].

Uganda lacked accurate data on the prevalence of birth defects which could guide prevention, care activities, and interventions for birth defects [4].

In this paper, we describe the implementation of the electronic surveillance system and the successes, challenges, and lessons learned from using mobile tablets for data collection in a hospital-based birth defects surveillance study in a resource-limited setting of Kampala, Uganda.

## Materials and methods

### Implementation of birth defects surveillance system

We implemented a hospital-based birth defects surveillance system in four hospitals in Kampala, Uganda: one public/government hospital (Mulago National Referral Hospital) and three faith-based, private, not-for-profit hospitals (Mengo Hospital; St. Francis Hospital, Nsambya; and Uganda Martyrs Hospital, Lubaga). We estimated that the surveillance system would capture approximately 50,000 deliveries per year [4], including live births, stillbirths, and spontaneous abortions, in the participating hospitals. We trained surveillance midwives to collect maternal demographic and health information (age, HIV status, obstetric history) and birth outcome information (mode of delivery, infant sex, gestational age, and results from infant examinations) for every delivery as shown in Table 1. This information had to be collected between the time of birth and discharge—usually within the first 24 hours after delivery. More details about this surveillance system are described elsewhere [4].

**Table 1:** List of data collected by category.

No.	Category	Variable name	Explanation and instructions
	***Introductory information***	Hospital	Indicate which hospital the mother gave birth
1		Identification Number	Indicate mother’s identification number(s)
2		Mother’s names	Write mother’s Surname, first name
3		Father’s names	Write Father’s Surname, first name
4		Tribe	Enter/Write mother’s tribe
5		Village	Enter/Write mother’s Village
	***Maternal Information***		
6		Mother’s age (Completed years)	Enter mother’s age (complete years she has lived) and or mother’s date of birth
7		ANC first visit date	Enter/Write mother’s date of ANC first visit in date, month year format (dd/mm/yyyy)
8		LNMP	Indicate mother’s Last Normal Menstruation Period date (dd/mm/yyyy)–This should not be less than 10 months from current date
9		Received ANC care	Indicate whether mother received ANC care and how many times
10		Referral	Indicate whether the mother was referred to this hospital for delivery of this particular pregnancy and reasons for referral
	***Obstetric History***		
11		Previous Pregnancies	Indicate total number of previous pregnancies and outcomes of each of the pregnancies
12		Birth defects	Indicate if mother has ever had previous deliveries with birth defects and the types of birth defects she gave birth to, if she ever had any
13		HIV	Enter/Write mother’s HIV status
14		ART	Indicate whether mother is on ART and regimen she is on if applicable
15		ART date	Indicate when mother started ART and source of ART data if applicable
	Fetus/Infant Information		
16		DOB	Indicate infant’s date of birth (dd/mmm/yyyy)
17		Delivery Type	Enter/Write type of delivery
18		Had Delivery complication?	**Indicate whether mother had any delivery complication**
19		Delivery complication	**Indicate Delivery complications:**
20		Sex	Indicate infant’s sex
21		Gestation age	Enter/Write infant’s gestational age and outcome of birth
23		Weight	Enter/Write infant’s weight in grams
24		Head circumference	Enter/Write infant’s head circumference for examined babies
25		Baby body length	Enter/Write infant’s body length
22		Multiple births	Indicate whether birth was multiple and specify timing
23		death	Indicate whether baby died soon after birth
		ENND date	**Enter Date of ENND**
24		Birth defect	Indicate whether baby has been born with a birth defect
25		Major external birth defects	Enter/Write if the baby has a major external birth defect after examination
26		Photo consent	Indicate whether mother has consented to take photographs of her baby
27		Photograph	Take 4 clear photographs of the baby from various angles as per photograph taking SOP
28		Birth defects	List all the external major birth defects present if applicable
29		Birth defects description	Enter/Write a full and conclusive but precise narrative description for each of the external major birth defects you have identified and listed above
30		ICD-10	Code the birth defects you have identified and described above
31	Comments	Comments	Enter/Write any general comments for the entire form
32		Birth defects confirmation	Indicate if birth defects identified above have been confirmed
33		Doctor’s Signature	Sign the form if you have reviewed the birth defect (doctor)
34		Completed by	Indicate who has completed the form
35		Date completed	Indicate date the form has been completed
36		QC1	Enter/Write employee ID of who has done QC1 for the form
37		Date QC	Enter/Write date QC has been done
**Case-control nested component**			
No.	Category	Variable name	Explanation and instructions
	Introductory information	Hospital	Indicate which hospital the mother gave birth
1		Identification Number	Indicate mother’s identification number(s)
2		Mother’s names	Write mother’s Surname, first name
3		Father’s names	Write Father’s Surname, first name
4		Tribe	Enter/Write mother’s tribe
5		Village	Enter/Write mother’s Village
	Maternal Information		
6		Mother’s age (Completed years)	Enter mother’s age (complete years she has lived) and or mother’s date of birth
7		ANC first visit date	Enter/Write mother’s date of ANC first visit in date, month year format (dd/mm/yyyy)
8		LNMP	Indicate mother’s Last Normal Menstruation Period date (dd/mm/yyyy)–This should not be less than 10 months from the current date
9		Received ANC care	Indicate whether mother received ANC care and how many times
10		Referral	Indicate whether the mother was referred to this hospital for delivery of this particular pregnancy and reasons for referral
	Obstetric History		
11		Previous Pregnancies	Indicate total number of previous pregnancies and outcomes of each of the pregnancies
12		Birth defects	Indicate if the mother has ever had previous deliveries with birth defects and the types of birth defects she gave birth to, if she ever had any
13		HIV	Enter/Write mother’s HIV status
14		ART	Indicate whether mother is on ART and regimen she is on if applicable
15		ART date	Indicate when mother started ART and source of ART data if applicable
	Fetus/Infant Information		
16		DOB	Indicate infant’s date of birth (dd/mm/yyyy)
17		Delivery Type	Enter/Write type of delivery
18		Had Delivery complication?	Indicate whether mother had any delivery complication
19		Delivery complication	Indicate Delivery complications:
20		Sex	Indicate infant’s sex
21		Gestation age	Enter/Write infant’s gestational age and outcome of birth
23		Weight	Enter/Write infant’s weight in grams
24		Head circumference	Enter/Write infant’s head circumference for examined babies
25		Baby body length	Enter/Write infant’s body length
22		Multiple births	Indicate whether birth was multiple and specify timing
23		death	Indicate whether baby died soon after birth
		ENND date	Enter Date of ENND
24		Birth defect	Indicate whether baby has been born with a birth defect
25		Major external birth defects	Enter/Write if the baby has a major external birth defect after examination
26		Photo consent	Indicate whether mother has consented to take photographs of her baby
27		Photograph	Take 4 clear photographs of the baby from various angles as per photograph taking SOP
28		Birth defects	List all the external major birth defects present if applicable
29		Birth defects description	Enter/Write a full and conclusive but precise narrative description for each of the external major birth defects you have identified and listed above
30		ICD-10	Code the birth defects you have identified and described above
31	Comments	Comments	Enter/Write any general comments for the entire form
32		Birth defects confirmation	Indicate if birth defects identified above have been confirmed
33		Doctor’s Signature	Sign the form if you have reviewed the birth defect (doctor)
34		Completed by	Indicate who has completed the form
35		Date completed	Indicate date the form has been completed
36		QC1	Enter/Write employee ID of who has done QC1 for the form
37		Date QC	Enter/Write date QC has been done

### Selection of electronic over paper-based data collection system

Before implementing the surveillance system, we held discussions with the study team and hospital midwives to decide between using paper forms or electronic data capture. We initially thought paper forms would be better because hospital staff were used to paper forms. However, since this surveillance project was designed to collect a high volume of data, it would be difficult to keep track of all the paper forms, make sure copies were always available, and transport forms from the sites to the study office for data entry. We decided to use tablets for data collection with paper forms used only as a backup because all study data collection forms could be built into the software with real-time data entry and checks, which would improve data completeness and accuracy. However, most hospital staff were not used to electronic data entry and had concerns about the feasibility of carrying the tablets from one ward to another when conducting routine nursing duties. Lastly, because of the lack of free accessible internet at these hospitals, transferring the information to the server was perceived to be an obstacle.

All midwives recruited to work on the study in the government hospital were computer literate, and those from other hospitals were trained to conduct surveillance activities [5]. Surveillance midwives at all hospitals carried the tablets in a crossbody bag so as not to hinder their nursing duties, to reduce possible damage to the tablet, and allow for easier movement from one ward to another.

### Software selection and programming

We chose Android tablets with a larger screen for better graphic display for collecting data because these were perceived as easy to use and were less expensive than other devices at that time. We conducted a literature search to identify a suitable software package that addressed our needs using the following criteria: (i) free and open-source, (ii) allows different forms of data collection (text, numeric, and media) to document birth defects with illustrations or photographs, (iii) allows offline data collection and later submission when the internet is available, (iv) ability to collect a large volume of data, and (v) able to run on Android devices.

We did a benchmark comparison between three popular systems at that time which included SurveyCTO, KoBoCollect, and ODK as shown in Table 2 below.

**Table 2:** Benchmark comparison of the three popular systems for data collection at that time.

Need item	Open Data Kit (ODK)	KoBoCollect	SurveyCTO
I. Free and open-source	Yes	Yes	No
II. Allows different forms of data collection	Yes	Yes	Yes
III. Collect a large volume of data	Yes	Yes	Yes
IV. Allows offline data collection	Yes	Yes	Yes
V. Able to run on Android devices	Yes	Yes	Yes

Both Open Data Kit (ODK) and KoBoCollect fulfilled the criteria. However, ODK software was selected because it fulfilled the criteria, supports a wide range of question and answer types, was popular and easy to use.

We programmed the study data collection tools into ODK with automatic validation and logic checks to ensure that any out-of-range data were identified in real-time. ODK also allowed for all data to be encrypted and safely stored.

### Data quality

We piloted the data collection activities to identify any programming errors before implementation [5]. Surveillance midwives entered data directly into the tablet, and another surveillance midwife/research assistant conducted quality control reviews (QC1) of the record. During QC1 checks, surveillance midwives/research assistants reviewed all variables entered into the tablet for consistency, wrong entries, missing values, and incorrect participant identifiers and cross-checked the information with the hospital patient file, and study surveillance delivery log before submission. Data were entered into the tablet in real-time, and logic checks and real-time quality control reviews were conducted daily to ensure that there were no missing data.

### Data security

**Fig 1:**
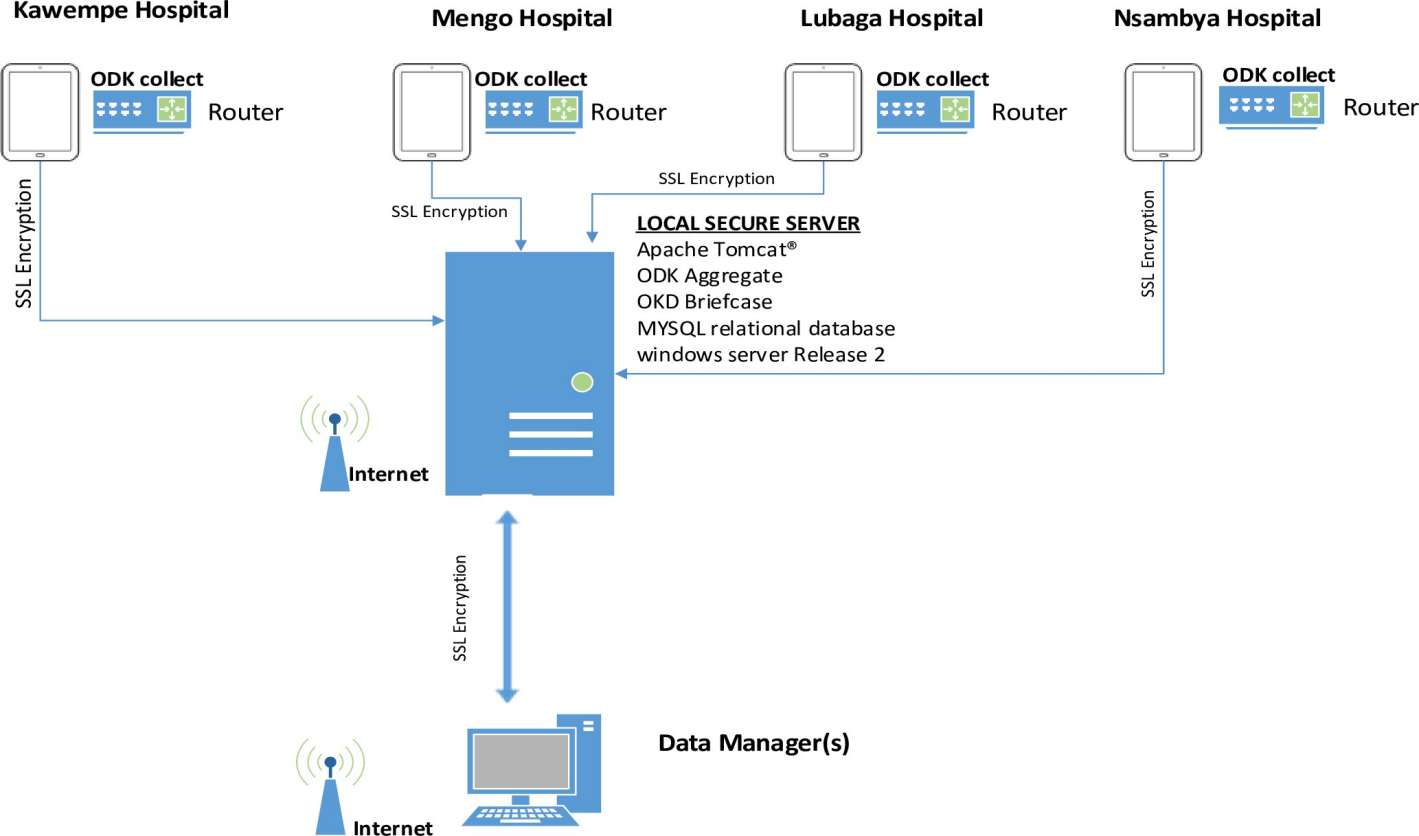
Data flow profile of the surveillance system from the four participating hospitals.

Before study initiation, all study staff and surveillance midwives signed an agreement regarding data safety and confidentiality, which included responsibility for the tablet. Also, all tablets were password-protected to prevent loss of data if the tablet was lost or stolen. Data were encrypted and securely transferred directly to the local server that was configured with a secure socket layer (SSL) key, Java (“Just Another Virtual Accelerator”) and Structured Query Language (MySQL) as shown in the data sharing frame work in Fig 1 below.

The surveillance midwives used password-secured internet from study routers, which were configured to be accessible only by study tablets. The internet password was only known by the data managers to prevent unauthorized access. Whenever there was an issue with the record after the data were submitted, the data manager would follow up with the surveillance midwife who collected the information to resolve the discrepancy and modify the database as needed.

### Ethics approval and consent to participate

This study was approved by the Joint Clinic Research Center (JCRC) Institutional Review Board, the Uganda National Council for Science and Technology (UNCST) and the US Centers for Disease Control and Prevention Institutional Review Board (protocol #6606).

Written informed consent was obtained from all mothers aged 14 years and above before taking photographs of the baby. For mothers aged under 14 years, both parental consent and assent were both obtained.

## Results

We implemented surveillance activities first at Mulago Hospital, a large government national referral and teaching hospital that has five delivery wards with approximately 30,000 births annually. Surveillance activities were piloted in the least busy ward so that we could identify and address any issues in study activities and data collection before moving to other wards and hospitals. The implementation of the study activities in other wards and hospitals was done in phases [4] after issues such as limited space to accommodate study activities were addressed [5]. Although we tested and piloted the data collection tools on the tablets, we experienced several issues when we implemented the surveillance system in the hospitals.

### Username and password for tablets

Sometimes the usernames and/or passwords that the surveillance midwives chose did not work; although in many cases we found that it was because the surveillance midwives forgot their usernames and/or passwords. We encouraged staff to choose usernames and passwords that they could remember easily (including capitalized letters or spaces). Staff were also advised to check with the research assistants or the data managers whenever they could not remember their passwords so that the data team could retrieve their information. Eventually, one username and password for each hospital was created and shared among surveillance midwives. The surveillance midwives were advised not to share it with anyone else to ensure confidentiality.

### Internet connectivity and data storage issues

Because of the slow internet or lack of connectivity, internet data bundles often expired. Some concrete buildings at all the hospitals had poor network connections, which affected uploading the forms to the central server. Inappropriate use of tablets for social media and video streaming also depleted the data bundles.

We used an internet tracking log to monitor the usage of the data bundles at each hospital so that data would not get depleted without our knowledge. Internet access was password-protected to limit access to only surveillance midwives using the study tablets to submit data, and the password was only known by the data managers. We uninstalled and locked some applications available on the tablet, such as Facebook and WhatsApp. We revised the study standard operating procedures (SOPs) to include the proper use of the tablets and the internet and then trained all staff on these SOPs. We estimated how many gigabytes of internet data each hospital required for 3 months. In cases of poor connectivity, we restarted or repositioned the router to improve network access. However, despite poor connectivity, the ODK software allowed study staff to collect data on the tablets and submit the data later when signal strength/internet was restored. In situations where the internet failed or if the data bundle was depleted, research assistants supported the surveillance midwives with their private internet bundles to ensure that data were submitted on time, and later the research assistants were reimbursed. We also switched to another internet service provider with better network strength at particular wards and hospitals.

### Tablet issues

The records took up most of the data space on the tablets, mostly because of the photographs documenting birth defects, which caused slow processing or freezing. The sensitivity of the tablet’s touch screen, working speed, and duration of the battery also decreased over time, which slowed the data collection process.

To increase the storage space and speed of the tablets, we backed up all photographs on the study server and deleted them from the tablets. We also uninstalled tablet applications that were not study-related, such as social media apps and games. To improve battery strength, we ensured that all tablets were fully charged daily by advising the midwives to always charge them. Surveillance midwives were advised to activate the Wi-Fi on the tablet only at the time of sending data and then disconnect it to reduce power consumption. We also replaced a few tablets that had weak batteries and poor touch screen sensitivity. We advised surveillance midwives to be patient while uploading the forms on tablets and to always report any issues to the research assistant or data manager. We also developed a tablet error correction form to document the identified tablet issues.

The study tablets were kept at the respective hospitals, and midwife supervisors were responsible for the tablets during their shifts. Midwife supervisors allocated tablets to surveillance midwives, maintained a tablet allocation log, ensured adherence to the tablet SOPs, and informed the data team whenever technical support or unscheduled maintenance was needed. Surveillance midwives completed a tablet tracking log, including date and time issued, tablet number, the tablet’s condition, and date and time returned. If a tablet was damaged, lost, or stolen, then the assigned user was responsible for the cost of repair/replacement as necessary. The midwife supervisor was responsible for verifying that the tablet was returned in good working condition before the surveillance midwife left at the end of the shift.

The midwife supervisor also was responsible for charging the tablets daily (or as often as needed) and for storing all tablets which are not in use at the hospital in designated locked storage spaces. They were also responsible for ensuring that the tablet accessories were not taken away from the study premises at any time. Midwife supervisors were not allowed to share the key to the storage space for the tablets; lost keys were immediately reported to the data manager.

Tablets were used by only authorized surveillance midwives who were required to always keep the tablets in their bags when not in use during their work shifts. Because surveillance midwives frequently moved from one ward to another, they could be targeted by thieves; therefore antitheft measures such as the cross bags were used to prevent theft [6].

### Data entry

We experienced several issues related to data entry. Sometimes barcodes that had been already assigned to another baby were scanned for a new entry. Surveillance midwives sometimes had difficulty holding the tablet and baby securely while taking pictures, and the high-speed tablet camera could inadvertently scan a barcode that had already been used while the surveillance midwives were positioning the tablet to scan a new barcode. If the error was not observed by the surveillance midwives, the incorrect barcode data were submitted, duplicating identifiers in the database.

Low-quality pictures were also a challenge. After obtaining the parental consent, surveillance midwives took photographs of infants with birth defects that would be reviewed by the birth defects experts. These photographs included at least one view of the newborn’s entire body and at least three focused views of the birth defect showing different side views or front/back views depending on the location of the birth defect. Some of these photographs were unfocused or had shadows because of lighting issues, which made it difficult for the birth defect expert to confirm the defect.

Sometimes forms could not be submitted because the system date on some tablets would automatically revert to the factory date, and when the system date differed from the entered dates (such as date of delivery), the data could not be submitted. Surveillance midwives could not immediately correct the tablet’s date because only data managers knew the password for the system settings.

To address data entry issues, we revised our data entry SOP to emphasize real-time quality control to identify and correct erroneous scanning, multiple assigning of barcodes, low-quality photographs, and incorrectly entered values. We conducted refresher training on how to take clear photographs that capture the required positions of the infant and birth defect. We collected regular feedback from the midwives regarding the quality of the data entry forms and usability of the tablets, which guided form modifications. We also added data input options and modified inbuilt checks in ODK whenever necessary. Surveillance midwives were retrained to conduct timely quality control on the collected data. Missed births and delays in entering data were reduced by increasing the number of surveillance midwives at the smaller hospitals to improve coverage. We also held discussions with the ward in-charge to make sure they included surveillance midwives on each shift as they made the schedules. The tablet passwords were shared with the research assistants, so they could quickly correct the tablet system date and time if it reset to the factory date.

### Data submission

We experienced connectivity issues when the study server was down because the SSL key would expire, so the data could not be submitted. We had to periodically monitor and renew our SSL certificate to ensure secure data transmission between the tablets and the local server with encryption. To reduce issues related to data transmittal, similar surveillance programs could obtain a longer SSL certification period. We also restarted the Apache-Tomcat version 6.0 program on the server. All wards were given study phones and airtime, so they could quickly alert the data managers if data submission failed and could still enter data on the tablets and submit later when the system was restored. Data managers only approved the use of paper forms when there was a prolonged system failure. Once service was restored, data were entered into a tablet and submitted. Paper forms were stored in locked cabinets. We also found that we lacked enough storage space on the study server because this surveillance project collected a large amount of data, and the same server was used by other studies.

Another issue we encountered was that some babies were documented more than once. This mainly occurred when the mother was transferred or after a shift change when midwives in different shifts examined the same baby. There were delays in data entry, conducting QC1, and missing participants because of the high workload in some wards at the large government hospital and because surveillance midwives were not scheduled to cover some of the shifts at the smaller hospitals.

### Staff training

We scheduled training and refresher training for the surveillance midwives to address monitoring and data collection errors that were identified by the data managers during the QC process. All surveillance staff from the four hospitals attended the same training in shifts on using tablets for data collection and the protocol. To ensure full shift coverage at the participating hospital, and to improve attendance at the training, we conducted the training in shifts of 5 days. Surveillance midwives were reimbursed for transportation costs for these full-day training. Also, we continuously provided training to hospital staff especially the new staff at the private/not-for-profit hospitals because staff turnover was high. We also provided refresher training on specific areas that were identified during the quality control processes; these training were incorporated in the periodic hospital performance meetings (usually quarterly). To promote team building, participants who are more proficient at using tablets help lead the training for those who do not have experience using tablets. We also trained a locum research assistant as a backup to support the large government hospital and to fill in at the other hospital if the research assistant was on maternity/sick/annual leave. The locum research assistant also could replace full-time research assistants in cases of departure to ensure a smooth transition.

## Discussion

We found that using mobile tablets for real-time data collection in a large hospital-based birth defects surveillance study in a resource-limited setting was successful. Although we encountered issues setting up the system, all four hospitals have continued to collect surveillance data electronically. From August 2015 to December 2018, information on 110,752 births was captured into the database of which only 0.2% (n = 179) were captured on paper forms. Photographs and illustrative diagrams were taken and successfully uploaded for each of the 1,044 babies identified with a birth defect. Extensive training and follow-up were needed to prepare staff, but the surveillance midwives are now able to collect study data with few issues.

To collect data for this surveillance study, we used free software, which is available in other resource-limited settings. However, our study was conducted in an urban center that likely has better internet connectivity than rural settings. We were unable to do a cost-effective analysis of the use of tablets compared to paper-based forms because we only used paper forms as a backup. However, paper-based methods have been reported to be costly compared to electronic systems [7].

One of the advantages of using ODK was that we were able to capture photographs of babies with birth defects as part of the electronic records. If a paper-based approach was implemented, it would be challenging to print, handle, and store the images without issues of loss of confidentiality or the potential for a mix-up of records.

Another advantage of electronic data capture was that the logic checks we implemented resulted in complete surveillance records because the midwives could not proceed from one data element to the next without entering a response, even if the response was “unknown” or “missing.” The system was also secure and reliable in cases of poor internet connectivity because data could still be collected on the tablets and stored until the internet was available. We did not experience any unauthorized use or theft of tablets, which we believe was due to accountability measures.

Using tablets for data collection improved performance because the data managers monitored collected data, identified errors quickly, and requested a resolution from the midwives then fixed and documented. Presentations addressing performance and areas for improvement were made during hospital meetings, and midwives were eager to learn about the quality of the data at their hospital.

## Conclusions

It is possible to use mobile tablets for real-time data collection for a hospital-based birth defects surveillance system in a resource-limited setting. However, there are several challenges at different stages of project implementation that will be encountered. Extensive training and follow-up can overcome challenges and are key to preparing staff for a successful data collection system. We instituted measures to overcome these issues and developed a smoothly running surveillance system that collects data on thousands of births each month. Also, similar surveillance programs should have a dedicated server with sufficient storage space for the large amounts of collected data Although our experience is limited to the participating Hospitals in an urban setting and generalizability is limited, lessons learned from our experience may be useful to other large-scale studies implementing electronic data collection. We also recommend future comparison between cloud base storage systems and on-premise storage systems since in our surveillance we only used the on-premise platform.
